# Were there royal herds? Understanding herd management and mobility using isotopic characterizations of cattle tooth enamel from Early Dynastic Ur

**DOI:** 10.1371/journal.pone.0265170

**Published:** 2022-06-15

**Authors:** Tina L. Greenfield, Augusta M. McMahon, Tamsin C. O’Connell, Hazel Reade, Chris Holmden, Alexandra C. Fletcher, Richard L. Zettler, Cameron A. Petrie

**Affiliations:** 1 Department of Archaeology and Anthropology, University of Saskatchewan, Saskatoon, Saskatchewan, Canada; 2 Department of Archaeology, University of Cambridge, Cambridge, United Kingdom; 3 Institute of Archaeology, University College London, London, United Kingdom; 4 The Saskatchewan Isotope Laboratory, Department of Geological Sciences, University of Saskatchewan, Saskatoon, Saskatchewan, Canada; 5 National Horse Racing Museum Palace Street, Newmarket, Suffolk, United Kingdom; 6 University of Pennsylvania, Near Eastern Languages & Civilizations, Philadelphia, Pennsylvania, United States of America; University at Buffalo - The State University of New York, UNITED STATES

## Abstract

During the third millennium BC, Mesopotamia (the land between the Tigris and Euphrates Rivers, in modern Iraq-Syria), was dominated by the world’s earliest cities and states, which were ruled by powerful elites. Ur, in present-day southern Iraq, was one of the largest and most important of these cities, and irrigation-based agriculture and large herds of domesticated animals were the twin mainstays of the economy and diet. Texts suggest that the societies of the Mesopotamian city-states were extremely hierarchical and underpinned by institutionalised and heavily-managed farming systems. Prevailing narratives suggest that the animal management strategies within these farming systems in the third millennium BC were homogenous. There have been few systematic science-based studies of human and animal diets, mobility, or other forms of human-animal interaction in Mesopotamia, but such approaches can inform understanding of past economies, including animal management, social hierarchies, diet and migration. Oxygen, carbon and strontium isotopic analysis of animal tooth enamel from both royal and private/non-royal burial contexts at Early Dynastic Ur (2900–2350 BC) indicate that a variety of herd management strategies and habitats were exploited. These data also suggest that there is no correlation between animal-management practices and the cattle found in royal or private/non-royal burial contexts. The results demonstrate considerable divergence between agro-pastoral models promoted by the state and the realities of day-to-day management practices. The data from Ur suggest that the animals exploited different plant and water sources, and that animals reared in similar ways ended up in different depositional contexts.

## Introduction

**Fig 1:**
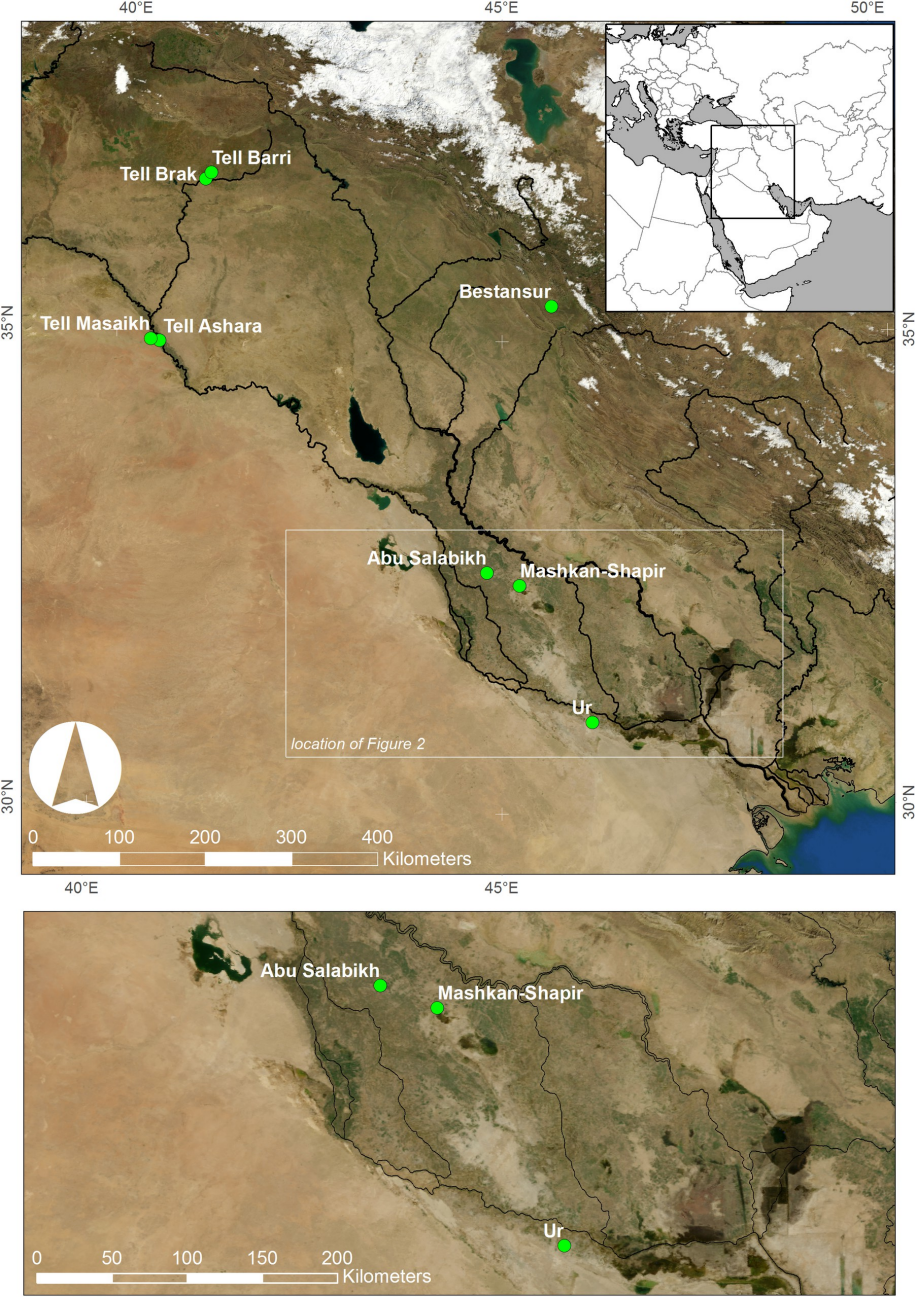
Map of Mesopotamia showing the location of sites mentioned in the text. Background imagery courtesy of NASA Blue Marble: Next Generation satellite imagery produced by Reto Stöckli and obtained from NASA’s Earth Observatory (NASA Goddard Space Flight Center) [see: http://earthobservatory.nasa.gov/Features/BlueMarble/]. Map composed by C.A. Petrie.

Ancient Mesopotamia, the land between the Tigris and Euphrates Rivers, stretches across modern Iraq and Syria and was home to the world’s earliest cities and states. During the Early Dynastic (ED) period (*c*.2900-2350 BC), city-states dominated the political and physical landscape of southern Mesopotamia, or ancient Sumer (Fig 1) [1,2]. Irrigation-based agriculture and large herds of domesticated animals were the twin mainstays of the region’s economy and diet. From *c*.2500 BC onwards (ED III Period), cuneiform text archives refer to agricultural and pastoral production and management, and to the people, goods, and other resources that supported the dense network of cities, towns and villages that dot the countryside [3]. However, Mesopotamian texts are neither comprehensive nor representative of non-elite society and economy during the ED III. Typically, texts reflected the interests and needs of the scribes and their employers (temple and palace), and not the illiterate majority of the population. Recent analyses of zooarchaeological data from other parts of the Near East have clearly demonstrated that textual sources relating to animal exploitation are extremely biased, even in later periods when literacy was more pervasive [4–9].

Scientific analyses of archaeological remains have demonstrated the utility of systematically studying osteological material recovered from excavations. The study of animal and human remains, including at a biomolecular level, can increase our knowledge of the daily practices and sustainability of early urban societies by examining factors such as: 1) human food economy and diet; 2) the flexibility of animal management strategies [10–13]; 3) social hierarchies and inequalities, resource access, consumption and distribution; and 4) human and animal mobility [14,15]. Most of the excavations at major sites in south Iraq, however, were carried out in the early twentieth century before scientific analyses were available, and the number of excavations that have incorporated archaeological science approaches has been limited [e.g.,^16^–^18^].

Scholars have explored the role of animals as documented in the Mesopotamian text corpus in some detail (e.g., *Bulletin for Sumerian Agriculture* volumes VII-VIII; [19: 70–89], but the limited zooarchaeological studies for southern Mesopotamia [20–25] mean that a wide range of questions remains unexplored. Isotopic analysis is increasingly used in archaeological research to address questions of mobility and consumption in both humans and animals, but its use in Mesopotamia has been limited to examining a small number of humans, animals, plants and soils [26,27]. This paper presents a study based on oxygen, carbon and strontium isotopic analyses measured in zooarchaeological material excavated from the site of Ur in the 1920s. These data provide insights into animal diet, herd management and animal movement. The specific aim of this paper is to identify the degree of similarity and/or variability in the diet, water consumption and mobility patterns of bovines (cattle) found in two types of context within the Royal Cemetery of Early Dynastic III Ur: royal and private/non-royal graves. The Royal Cemetery at Ur contained a range of different types of burials, including a number that were large, elaborate and included rich grave goods and in some instances evidence for human and animal sacrifice, and were designated and described as “royal” by the excavator, Leonard Woolley [28–30]. Other graves were designated as ‘private’ or non-royal, and while some of these contained rich grave goods and may have held non-royal elites, many had few or no grave goods at all. The resulting data make it possible to explore larger questions of animal management and short-range mobility, as well as ascertaining whether certain animals were reserved for those buried in the more elaborate “royal” tombs and were treated differently from animals buried in private/non-royal graves.

## Context

Recent research has demonstrated that the alluvial plains of south Mesopotamia were more ecologically diverse than previously acknowledged [31,32]. It is now clear that there were environmental micro-regions with variable resource clusters and differential water access, and these differences have implications for regional economies and land use normally invisible in the texts [31,32]. The earliest Mesopotamian cities belonged to integrated settlement systems marked by interdependence among cities, towns, villages, and pastoralists for sustenance, but there appears to have been a shift toward city-based farmers and pastoralists in the early-mid third millennium BC [33: 141–155, 34: 72–75, 35: 53–57, 59–60, 36: 141–144, 37: 329–330] see also [19: 56–90]. However, these reconstructions are based on settlement distribution data, and we lack a nuanced picture of the political economy of cities, particularly in terms of the ways that elite (royal and/or higher status) and non-elite (non-royal and/or lower strata) citizens interacted with the animal populations of the city, and how both people and animals moved within their local landscape. The texts mainly record the animals that fed and supplied secondary products to the palace and temple institutions [38]. These herds were dominated by sheep and goats and, to a lesser degree, bovines (domestic cattle) [39]. Notably, privately owned animals such as pigs, and hunted wild animals rarely appeared in texts, but are present in the archaeological record [24]. There remain many questions about the management of these herds, particularly the sources of their food and water, as well as their degree of mobility and whether they moved across short, medium or long distances.

## Ur and The Royal Cemetery

**Fig 2:**
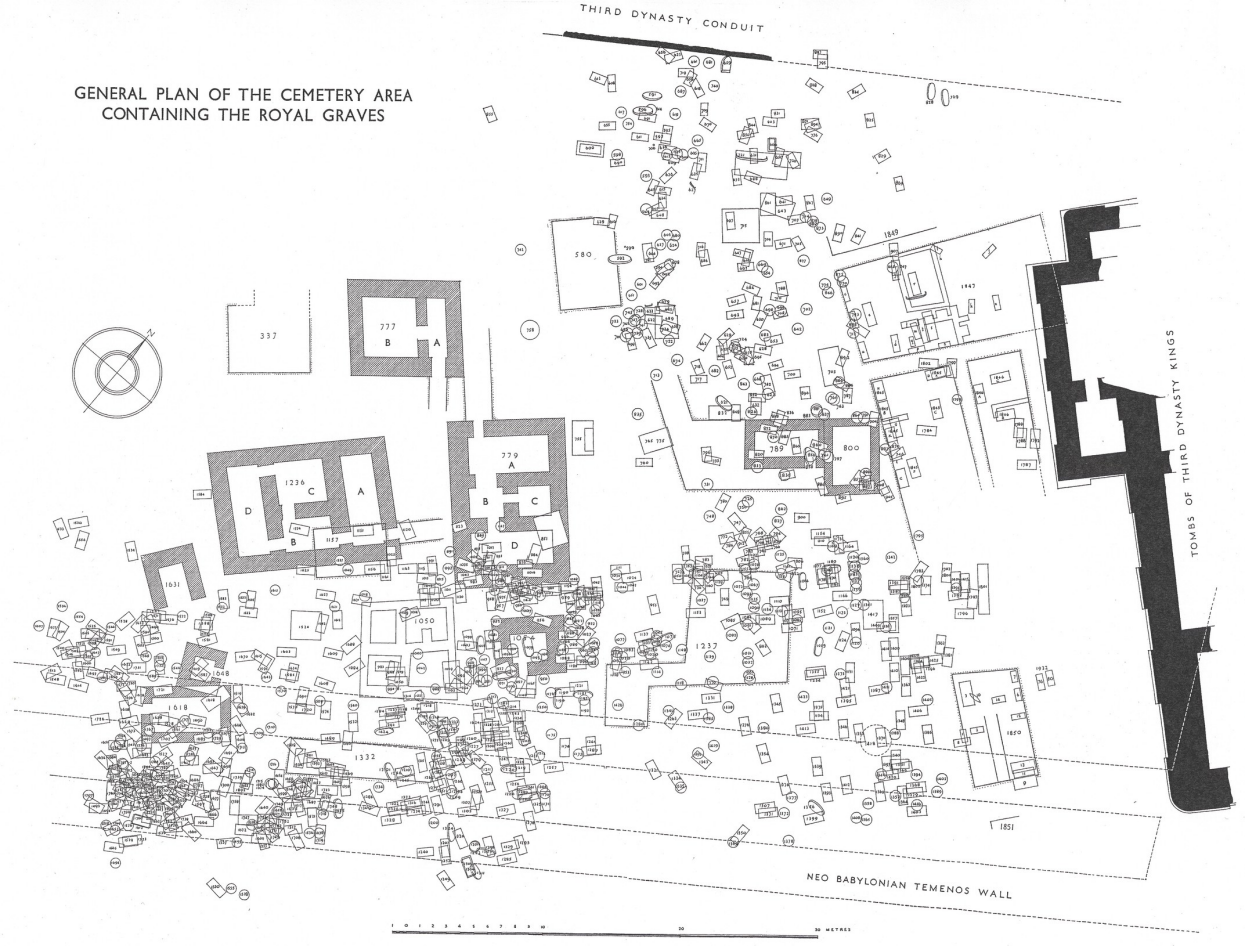
Layout of the Royal Tombs and private/non-royal graves within The Cemetery (Ur-online.org cf. Woolley 1934: Vol. II Plates 273 and 274). Republished from [Ur-online.org cf. Woolley 1934 Vol. II Plates 273 and. 274] under a CC BY license, with permission courtesy of the Penn Museum original copyright 1934, images 142563 and 149980.

During the Early Dynastic period, Ur developed into a major urban centre, reaching at least 50 hectares in extent, and it was the centre of one of the largest and most important of Mesopotamia’s city-states, [40]. The city lay on the Euphrates River and near the head of the Persian Gulf and was situated within prime agricultural land, close to extensive areas of marshes and steppe suitable for grazing. During the Early Dynastic period, Ur developed a strong hereditary leadership and several temple institutions. Texts suggest that both institutional and privately-owned animal herds were present in and around the city, and it was extensively interconnected within southern Mesopotamia through a network of canals [41–43]. The Royal Cemetery (Fig 2) was located near the centre of the city and contained at least 2000 burials, dating to the mid- to late third millennium BC. The earliest graves (c. 2600–2500 BC) contained the burials of kings, queens, and other elites, but the area was subsequently used for non-royal burials, including within the ED III Period [28]. A small number of the royal burials were accompanied by human sacrifices that suggest these rulers’ absolute power. Further, several of the royal graves contained cattle that appear to have been sacrificed at the time of burial [28], a practice which would have dramatically increased the relative cost and prestige of these burials, compared to the majority of burials in the cemetery where only portions of cattle or other animals were deposited as grave goods. Animal prices varied with seasonal and environmental change, but the cost of cattle and bulls was generally high, reflecting the investment in their breeding and their value for traction, secondary products and meat [see 44] for examples from the late 3rd millennium BC Ur III Period. The presence of sacrificed cattle in several private/non-royal graves in the Royal Cemetery indicates that this practice was not limited to royalty, but raises the question of whether a difference was nonetheless maintained between such graves through varying treatments of the cattle, thus giving them different values. The presence of a few selected body parts versus an entire cattle skeleton (only found in the royal graves) would suggest it was a question of relative privilege and scale rather than presence or absence of cattle within graves.

## Diet and mobility

Investigating diet and mobility at third millennium BC Ur allows for a broader understanding of the societal fabric and daily life in southern Mesopotamia. Due to its location adjacent to several different environmental zones (steppe, irrigated alluvial plains, marshes) during the Early Dynastic period, Ur had the potential to support several different dietary and herd management strategies [24,42]. Isotopic analysis makes it possible to explore how humans and animals moved within and between settlements beyond what was known from texts [45–52]. While there are scientific studies on human and animal mobility from other geographical regions, there are relatively limited data from Mesopotamia relevant to this issue (e.g., [26,27,53]). The environmental micro-regions of southern Mesopotamia, with resource clusters and differential water access, offered choices for strategies of herding and farming [32,40,53,54].

In Mesopotamia, cattle/bovines (typically *Bos taurus*) were exploited mainly for dairy products, manure, and traction, for both ploughing and transport, and less often for meat [55–58: 148]. Hollow ways (ancient tracks) suggest that cattle mainly moved only short journeys from cities to sources of water and fodder and back [58: 150]. The degree to which bovines were moved long distances *within* Mesopotamia is unknown, but zooarchaeological evidence shows that some bovines were moved very long distances from other regions. Domestic water buffalo (*Bubalus bubalis*), for example, first appeared in southern Mesopotamia in the mid-late third millennium BC, and zebu (*Bos indicus)* appeared soon afterwards [19: 254–9, 59–62], and both species were initially domesticated in the Indus River Basin in South Asia and must have moved or been transported from there [61,63,64].

## Approach

Isotopic time-series analysis of tooth enamel can provide life history information at sub-annual resolution. Strontium isotopic composition reflects the underlying geology upon which the animal fed [65]. Carbon isotope values reflect the isotopic composition of the animal’s diet, while the oxygen isotopic composition of enamel reflects that of drinking and dietary water over the period of enamel mineralization [66–68]. Hypsodont teeth of most herbivores form over a period of several months to a year, and the enamel mineralization process is progressive, from the tooth’s occlusal surface to the root. Down-tooth variation in isotopic composition can be used to examine seasonal variations in diet, drinking water, and landscape use, which can provide insight into seasonal herding strategies [49,50,66], although it is also recognized that tooth mineralization damps the recording of isotopic time-series signals relative to their input [69,70].

## Strontium isotopic variation

**Fig 3:**
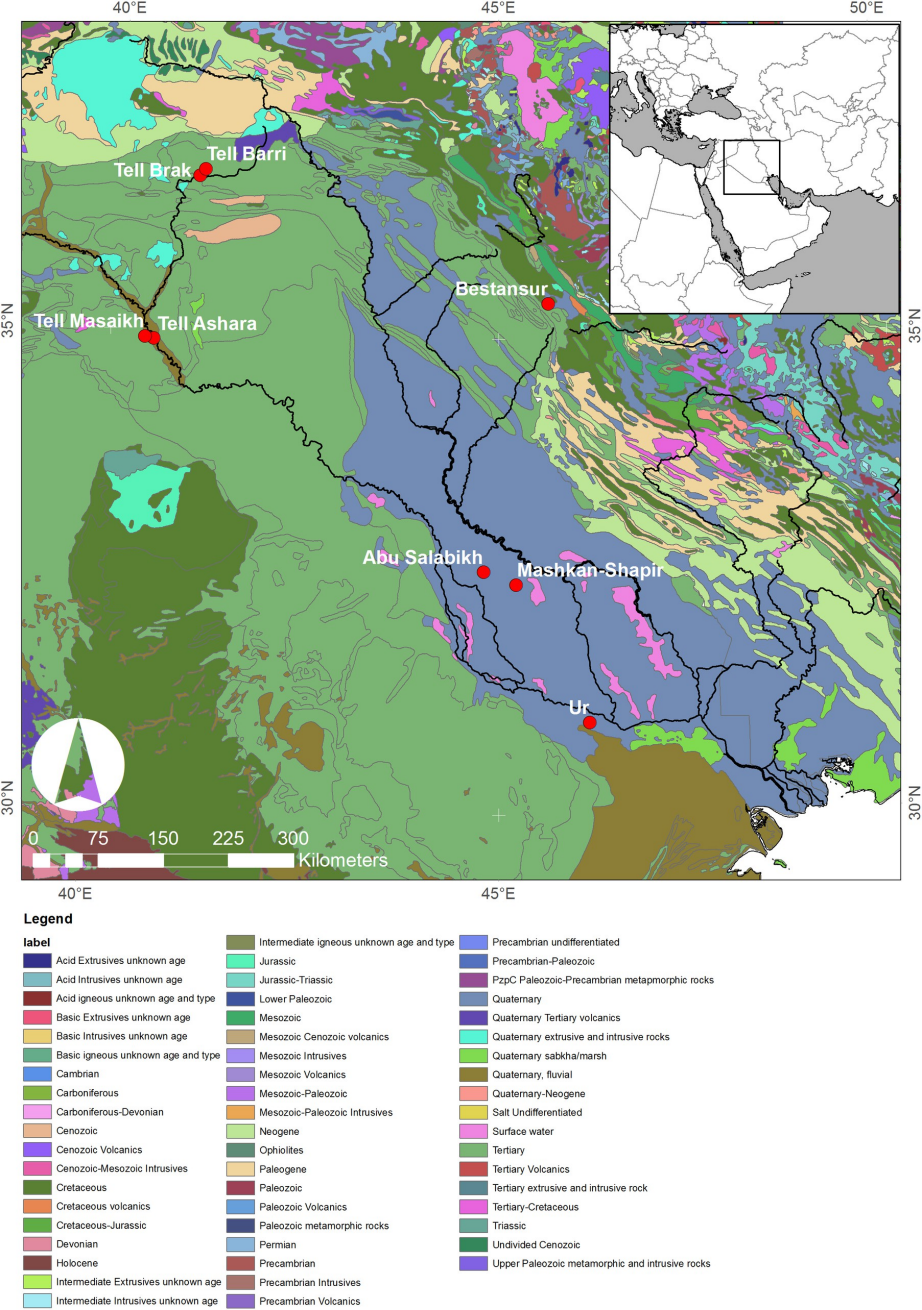
Map showing the geological substrate of Iraq and the location of sites mentioned in the text. World Geologic Map data for the Arabian Peninsula and Iran courtesy of the U.S. Geological Survey. Map composed by C.A. Petrie.

Tooth enamel strontium isotope ratios (^87^Sr/^86^Sr) reflect local geology, as strontium enters the food chain from soils and water. ^87^Sr/^86^Sr varies in rocks of different ages and of different types and can thus be used as a tracer of movement of animals across landscapes with different bedrock geology [65]. Animals reared in different settings may have different ^87^Sr/^86^Sr ratios, reflecting local-scale releases of Sr from soil mineral weathering. Strontium chemistry is similar to that of calcium. Strontium is soluble in water, taken up by plants ingested by the animals and preserved in their teeth and bones. Accordingly, animals reared at the same ancient settlements where their remains are found should preserve the environmental ^87^Sr/^86^Sr record of that site in the enamel of their teeth, whereas animals reared elsewhere will potentially record different ^87^Sr/^86^Sr ratios, provided that the soils are formed from isotopically distinct parent material [14]. As Ur is located in the large alluvial basin of the lower Tigris-Euphrates river system, the local soils will have an homogenised ^87^Sr/^86^Sr signal that is produced by the mixing of heterogeneous bedrock parent material during erosion and transport from elsewhere in the catchment. As such, little variation in tooth enamel ^87^Sr/^86^Sr is expected in animals that were raised in any proximity to Ur (Figs 1 and 3), and only animals transported long distances to the city could be expected to display differing ^87^Sr/^86^Sr.

## Oxygen isotopic variation

The ^18^O/^16^O ratio of an animal’s tooth enamel (presented as δ^18^O value) reflects the δ^18^O of water consumed either directly or in food, which is an integrated record of local and global oxygen isotope patterning. The δ^18^O value of precipitation at a given location is related to the latitude, altitude, distance to coast, amount of precipitation, humidity/aridity, surface air temperature and season, all of which contribute to spatially explicit patterns of δ^18^O in precipitation in the modern environment [71]. The δ^18^O of the local meteoric waters are modified through processes of evaporation, condensation and homogenisation in response to environmental parameters such as air temperature, amount of precipitation, residence time, and hydrological dynamics [72,73]. In southern Mesopotamia, water sources include surface water originating from rivers, canals, seasonal lakes and marshes, and groundwater originating from wells and natural springs [32,35: 71–99]. If water from surface sources like rivers or canals is consumed, there is likely greater isotopic seasonal variation than if water is consumed from groundwater sources, such as wells [74–77]. If animals drank from different water sources or consumed plants grown under different conditions, this may be recorded as differences in tooth enamel δ^18^O, either within or between individuals.

## Carbon isotopic variation

Tooth enamel ^13^C/^12^C ratios (presented as δ^13^C values) are controlled by the δ^13^C of the foodstuff being consumed during the time of tooth formation. δ^13^C values are used to distinguish between animal consumption patterns of two different types of plants, C_3_ and C_4_, which use different photosynthetic pathways [78]. Most plants use the C_3_ pathway, including economically important crops such as wheat, barley and legumes, as well as a range of wild plants and reeds, while a smaller group of plants use the C_4_ pathway, including domesticates such as millet and sorghum (not present in third millennium BC Mesopotamia), and wild plants adapted to arid conditions [79,80]. CAM (Crassulacean acid metabolism) plants are rare in Mesopotamia, and although some cactus species or other desert succulents with this pathway may have grown in the region, they are unlikely to have been used as reliable animal fodder [81]. The analysis of the δ^13^C in the Ur cattle provides information on individual animal diets and variability in diets between animals.

## The samples

**Fig 4:**
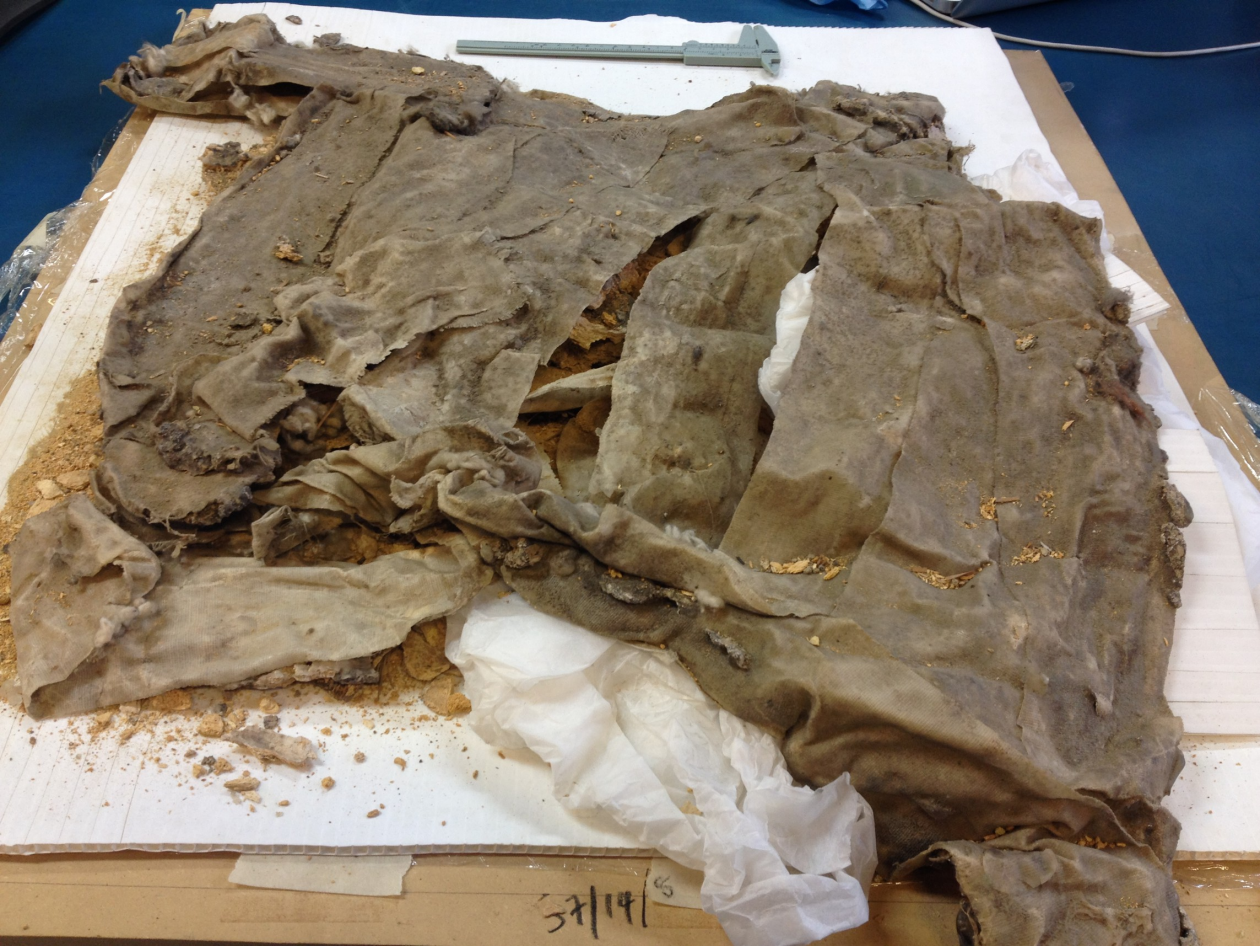
Cattle specimen BM1 (1935.01.16,16) “tooth 6” from PG789 “The Kings Grave” (Greenfield 2014).

**Fig 5:**
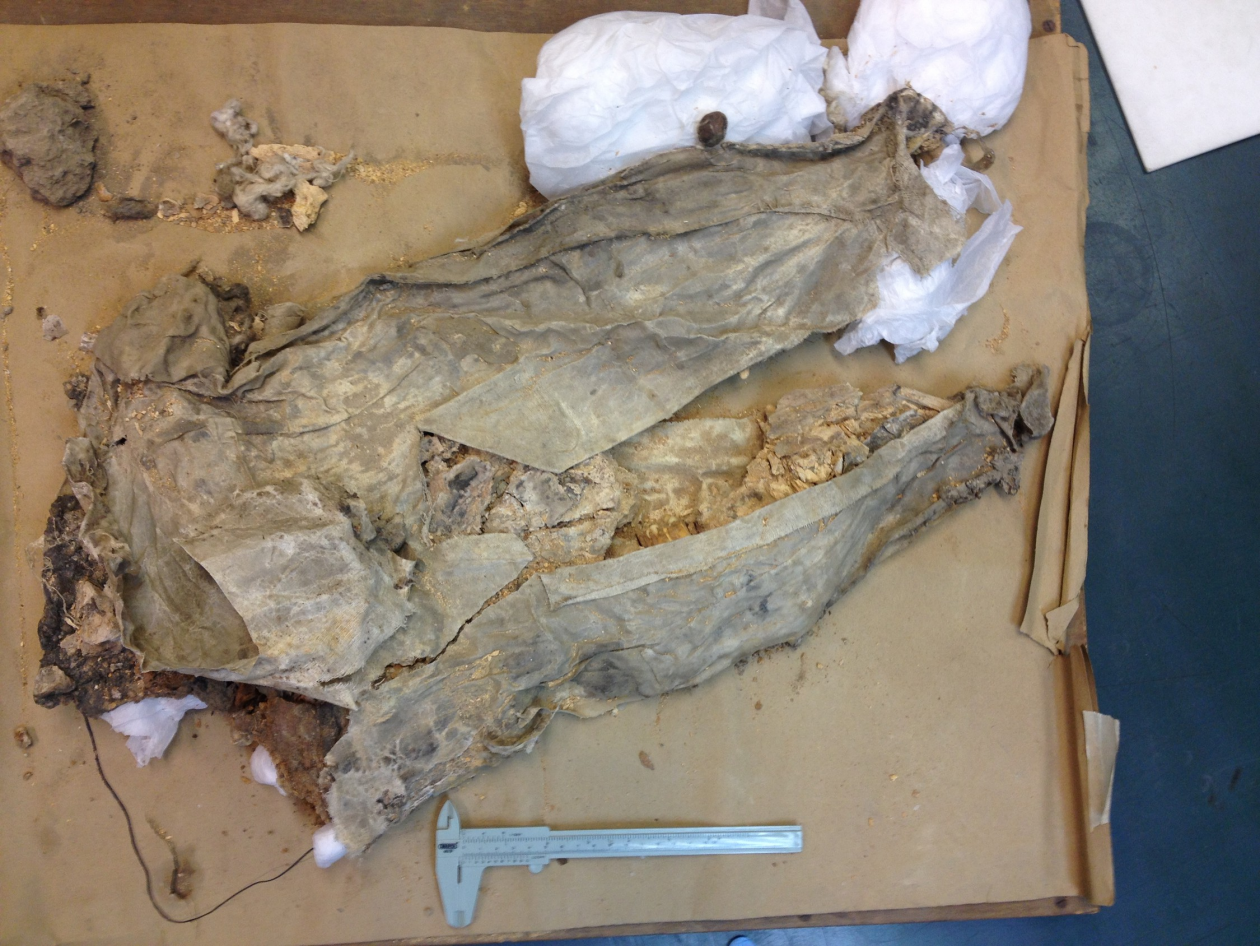
Cattle specimen BM2 (1935.01.16,17) “tooth 8” from PG789 “The Kings Grave” (Greenfield 2014).

**Fig 6:**
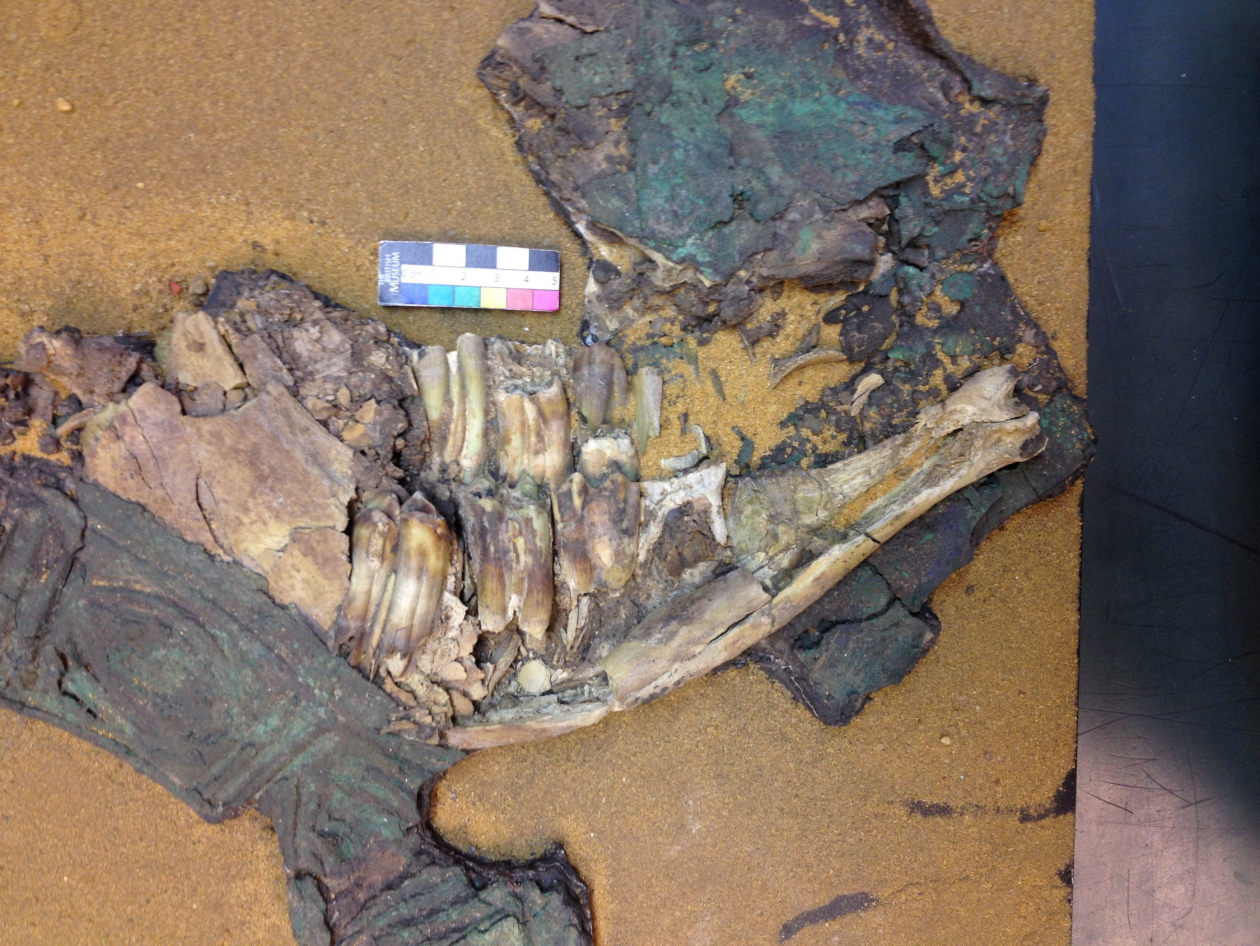
Cattle specimen BM3 (1928.10.10,164) PG800 Queen Pu-abi’s Grave. Note the harness attached to the animal still in place when retrieved by L. Woolley (Greenfield 2014).

Teeth were sampled from specimens of cattle (*Bos taurus*) collected during Leonard Woolley’s excavation of both royal and non-royal graves in the Royal Cemetery at Ur (see SI). Sample selection was limited by a range of factors, including preservation. Eight tooth specimens were chosen for isotopic analysis based on completeness, condition, temporal designation and, when available, secure provenience and contextual information (Table 1). Each specimen was a molar tooth (M1-3), which forms in bovines from approximately 6 months to 3 years. All samples were aged in order to give the determination of subadult (c. 3–4 years of age) for each individual. The animals were all killed during the prime of life and presumably chosen as high-status (conspicuous consumption) grave goods. Samples BM 1–4 all came from graves designated as royal by Woolley. It was thought that two samples (BM-1 and BM-2) came from the same individual located in PG789 “The King’s Grave”, but we suspect BM-1 (1935.1.16,17 ’tooth ’6’) (Fig 4) actually comes from a different burial (1935.01.16,16) within 789 because it was found lying on top of the support bandages around 1935.01.16,17 (see Fig 5), and there are also differences in the wear and root development of this tooth in comparison to BM-2 (see below). Sample BM-3 came from burial PG800, which was originally thought to be part of Queen Pu’abi’s Grave chamber (PG800B), but is now thought to be an additional Royal Tomb cut into the top of Queen’s burial complex [30,82–84] (Fig 6). BM-4 was used to provide additional ^87^Sr/^86^Sr data, but was not included in the δ^13^C and δ^18^O analyses. Samples NHM-1, NHM-2, NHM-7 and NHM-8 were all from graves designated as non-royal by Woolley; it must be acknowledged that their location within the same cemetery as graves of royalty suggests that these may have been burials of upper class members of society, but the lack of inscriptions and smaller numbers of grave goods indicate a status below royal (see SI for further contextual information and figure (S6 Fig) of before and after photos of the NHM specimens).

**Table 1:** Summary of *Bos taurus* teeth selected for sequential enamel δ^18^O, δ^13^C and ^87^Sr/^86^Sr analysis.

Sample Code	Curating Institute	Museum Specimen Identifier	Archaeological Context	Tooth
BM-1	British Museum	1935.1.16,17 ’Tooth 6’	PG 789 ’King’s Grave’	Lower right M_3_
BM-2	British Museum	1935.1.16,17 ’Tooth 8’	PG 789 ’King’s Grave’	Lower left M_3_
BM-3	British Museum	1928.10.10,164	PG 800 Pu-abi’s Grave	Lower M_2_
BM-4	British Museum	1935.1.16,19	PG 789 ’King’s Grave’	Upper M_3_
NHM-1	Natural History Museum, London	-	Burnt Layer above PG1054	Upper M_1_/M_2_
NHM-2	Natural History Museum, London	-	unknown PG	Lower M_3_
NHM-7	Natural History Museum, London	-	PG15	Lower M_3_
NHM-8	Natural History Museum, London	-	PG144	Upper M^2^

The ^87^Sr/^86^Sr signature of bioavailable Sr in southern Mesopotamia is poorly characterised, so we analysed twelve molluscs from 3rd millennium BC contexts (six *Galeommatoidea sp*. [bivalve] and one Gastropoda *Melanopsis Costata* [terrestrial snail] from Ur, and *Galeommatoidea sp*. (bivalve) from Abu Salabikh, situated in the centre of the south Mesopotamian alluvium c. 170 km NW of Ur), to provide baseline data [cf. 85]. Molluscs have been used as proxies to determine local ^87^Sr/^86^Sr ratios, since they provide data on local waters to compare to data for herded animals ingesting plants and water from the region or beyond [86].

## Results

Of the eight bovine teeth analyzed, all yielded ^87^Sr/^86^Sr data, and seven produced δ^13^C and δ^18^O data (see S1 Table). Twelve molluscs gave ^87^Sr/^86^Sr data. We contextualised our results by comparison to other published ^87^Sr/^86^Sr data from across the Mesopotamian basin, including results from two humans from the Royal Graves at Ur and three archaeological caprines (sheep/goat) from Mashkan-Shapir, which lies c. 180km NNW of Ur in the northern part of the Mesopotamian floodplain (Figs 1 and 3) [27]. Comparative material also included samples of plants and soil from various locations in Syria [87], humans from Tell Ashara in Syria [88], and plants from Bestansur in the Kurdistan region of Iraq [26] (Figs 1 and 3).

The ^87^Sr/^86^Sr results from this study suggest some variability in the regions where animals were reared, while the δ^18^O and δ^13^C results indicate variation in access to food and water resources between animals. Importantly, there is no clear division in resource access between cattle from royal and non-royal contexts.

### Strontium

**Fig 7:**
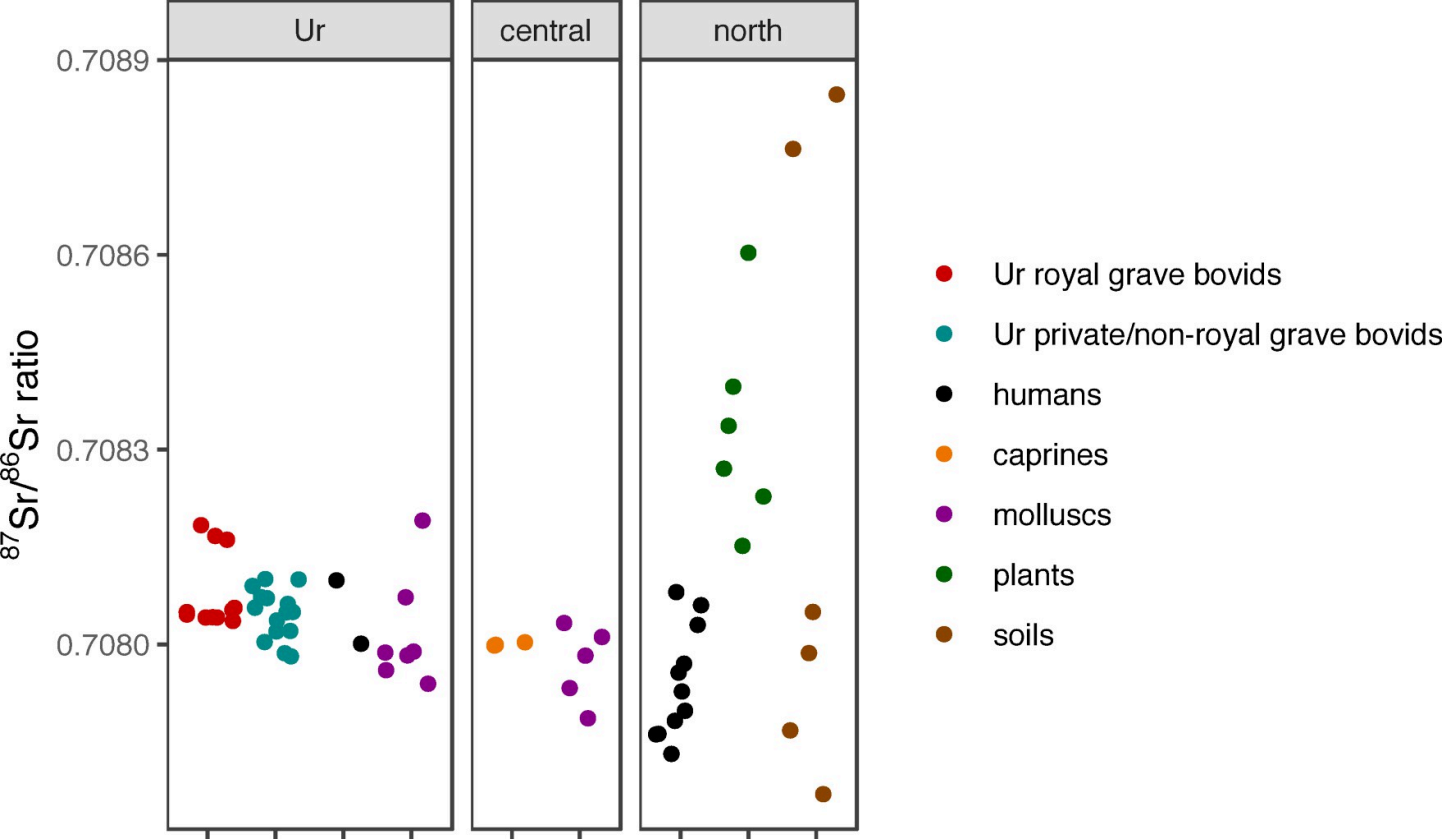
(animal_Sr4.eps): Enamel strontium isotope results from the bovines from Ur compared to those from humans, caprines, molluscs, plants and soils from the Mesopotamian region. See text for full details.

The cattle tooth enamel ^87^Sr/^86^Sr ratios from this study varied from 0.7080 to 0.7082, with seven in the lower part of the range and one with higher values (Fig 7). These are compared to other strontium isotope data from the region (Fig 7). The mollusc samples from Ur yielded ^87^Sr/^86^Sr ratios between 0.7079 and 0.7082, while those from Abu Salabikh spanned 0.7078–0.7081. The ^87^Sr/^86^Sr ratios of the human samples from Ur and the caprines from Mashkan-Shapir were 0.7080–0.7081 (Fig 7) [27], which are within the range measured for non-provenanced Iraqi dust (0.708–0.709) and archaeological glass from Mesopotamia (0.7081–0.7085) [89–91]. The ^87^Sr/^86^Sr ratios from soil and plants from various sites in northern Mesopotamia (Syria) are extremely variable, ranging from 0.7078–0.7088, while that of the humans from Tell Ashara ranges from 0.7079–0.7081 [88].

**Fig 8:**
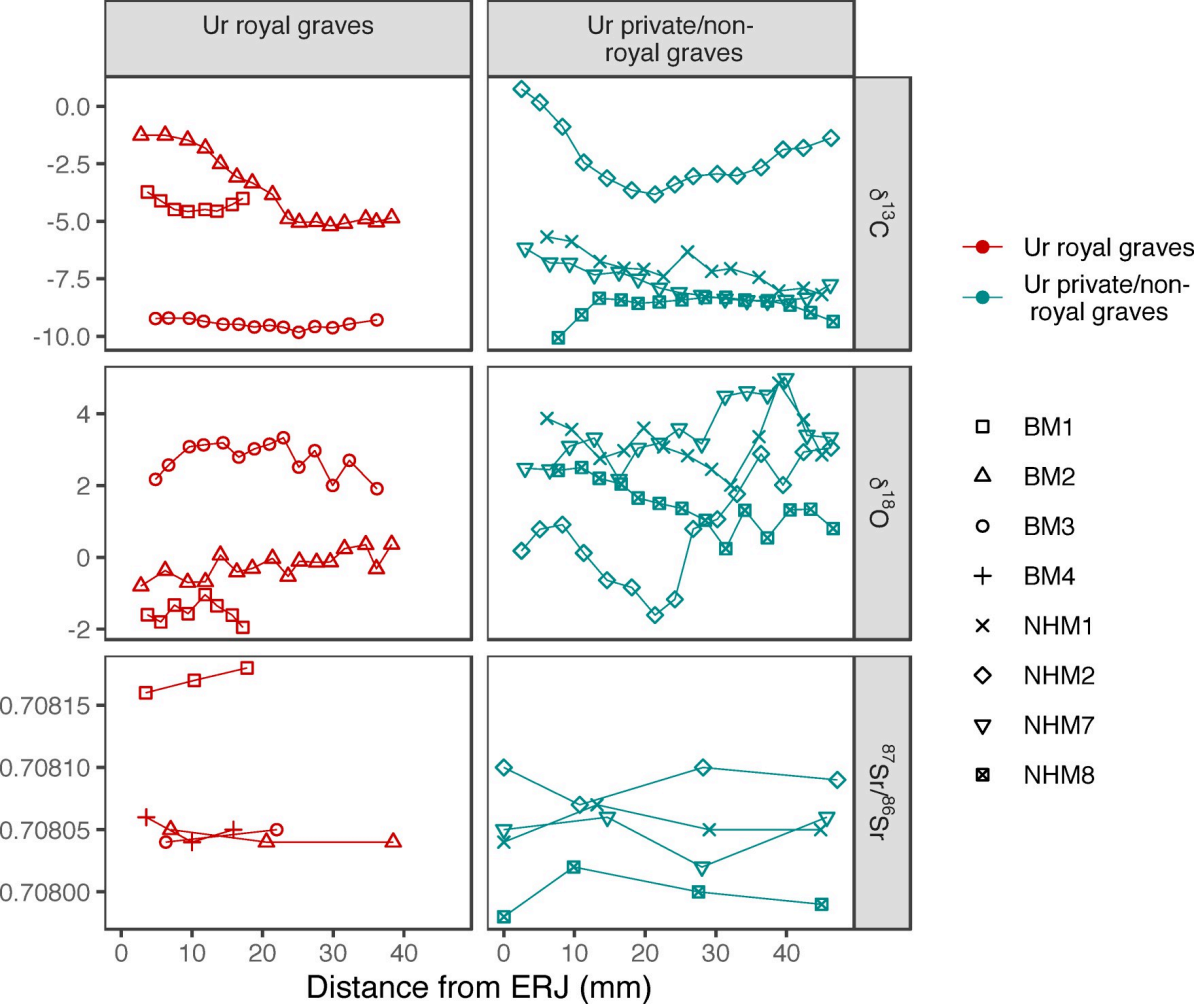
(intrateeth_COSr_labels.eps): Bovine enamel sequential intra-tooth carbon, oxygen and strontium isotope results plotted as a function of distance from the enamel root junction (ERJ), separated by individual (different shapes) and the royal/non-royal graves (different colours).

The ^87^Sr/^86^Sr results from the eight bovine teeth analysed for this study are consistent with seven of the animals being local to Ur, whilst the eighth (BM-1) has an isotopic signature that indicates an origin in a region with slightly higher bedrock ^87^Sr/^86^Sr, but potentially still somewhere within the greater Mesopotamian alluvial plain or the neighbouring piedmonts and uplands of the Zagros Mountains. The origin of BM-1 may be as far away as north Syria if the plant ^87^Sr/^86^Sr results from Tell Brak and the Balikh valley are a reliable indicator of local signals (Fig 7). Terrestrial environments that are close to coastal areas often receive wet and dry deposition containing strontium from seawater, that can drive bioavailable Sr towards the uniform ^87^Sr/^86^Sr ratio of seawater 0.7092 regardless of the underlying geology [92,93]. However, there is no compelling evidence for marine strontium inputs into the environments being used for rearing these cattle, as the signatures are typical of the narrow range of ^87^Sr/^86^Sr ratios that characterize the Mesopotamian alluvial plain more broadly (0.7080–0.7082) [27]. Mixing between terrestrial and marine sources would not be expected to generate such a such a narrow range of ^87^Sr/^86^Sr ratios over such a large region. There is a relatively uniform signal within each tooth, which shows that the sampled cattle at Ur were not moving between isotopically different settings on a seasonal basis within the early (late subadult/early adult) years of their lives (Fig 8). There is no ^87^Sr/^86^Sr difference between cattle in graves designated as royal and non-royal. These ratios are similar to the ^87^Sr/^86^Sr ratios reported from the people from Ur and the caprines from Mashkan-Shapir (Fig 8) [27]. The clear difference between the ^87^Sr/^86^Sr ratios of samples BM-1 and BM-2 confirms that they cannot have come from the same animal, which fits with the information on wear and on recovery context location mentioned above.

The strontium isotopic data for this study indicate that the eight tooth samples from Ur were from different animals, and at least seven of the eight animals were probably raised and lived on the southern Mesopotamian alluvial plain, with little evidence for movement to environments with different bedrock geologies within the lifetimes of these animals. The remaining animal while still from the alluvial plain could potentially come from as far away as north Syria or potentially to the east in Iran than the others.

### Carbon and oxygen

Tooth enamel δ^13^C values range from -10.1‰ to +0.7‰. The data indicate different proportions of C_3_ and C_4_ plants in the diets, both between different animals and during different seasons, clustering into two groups (Fig 8). Four of the teeth, BM-3 (from the royal burial context PG800) and NHM-1, NHM-7, and NHM-8 (non-royal burials), have δ^13^C values between -10.1‰ and -5.7‰, with intra-tooth range from 0.6‰ to 2.5‰, though with no clear seasonal patterns. Modern large herbivore tooth enamel *δ*¹³C is typically higher than dietary intake by approximately 14.1 ± 0.5 ‰ [94,95], so an estimation of *δ*¹³C dietary intake of -24 to -19.8‰ can be made, indicating that a very high proportion of the diet of these animals was composed of C_3_ plant species (see below). In contrast, BM-1 and BM-2 (royal burials) and NHM-2 (non-royal burial) all have higher δ^13^C values, ranging from -5.2‰ to +0.7‰. Two of these teeth (BM-2 and NHM-2) display large intra-tooth ranges of 0.9‰ and 4.6‰, respectively; the low intra-tooth range (0.9‰) of BM-1 in this instance most likely indicates a record that has been truncated by tooth wear, as the crown height available for sampling was considerably smaller than for the other specimens. Dietary estimates from these three teeth indicate a significant proportion of C_4_ consumption (see below), which varied considerably on a seasonal basis.

The δ^18^O results span -2.0‰ to +5.0‰, and mean animal δ^18^O ranges from -1.5 ± 0.3‰ (BM-1) to +3.5 ± 0.8‰ (NHM-7) (Fig 8). The smallest intra-tooth δ^18^O range is, as for the δ^13^C, observed in BM-1 (0.9‰), which again most likely indicates that the true variation has been lost due to tooth wear. The δ^18^O results from the royal and non-royal samples show considerable overlap, and within the royal and non-royal groups, differences occur between different animals. There is little consistent intra-tooth variation, with no clear seasonal patterns. This variation indicates that different animals ingested different water/food resources. The tooth enamel δ^18^O can be converted to meteoric water δ^18^O values via known relationships (from carbonate to phosphate [96]; from phosphate to meteoric water in domestic cattle, [97]). The tooth enamel δ^18^O values equate to δ^18^O_meteoricwater-VSMOW_ values of between c. -5.1 to +1.9‰, which encompasses most of the range of published modern groundwater and precipitation δ^18^O for southern and eastern Iraq (-6 to -1‰), but also includes higher values [98–100].

**Fig 9:**
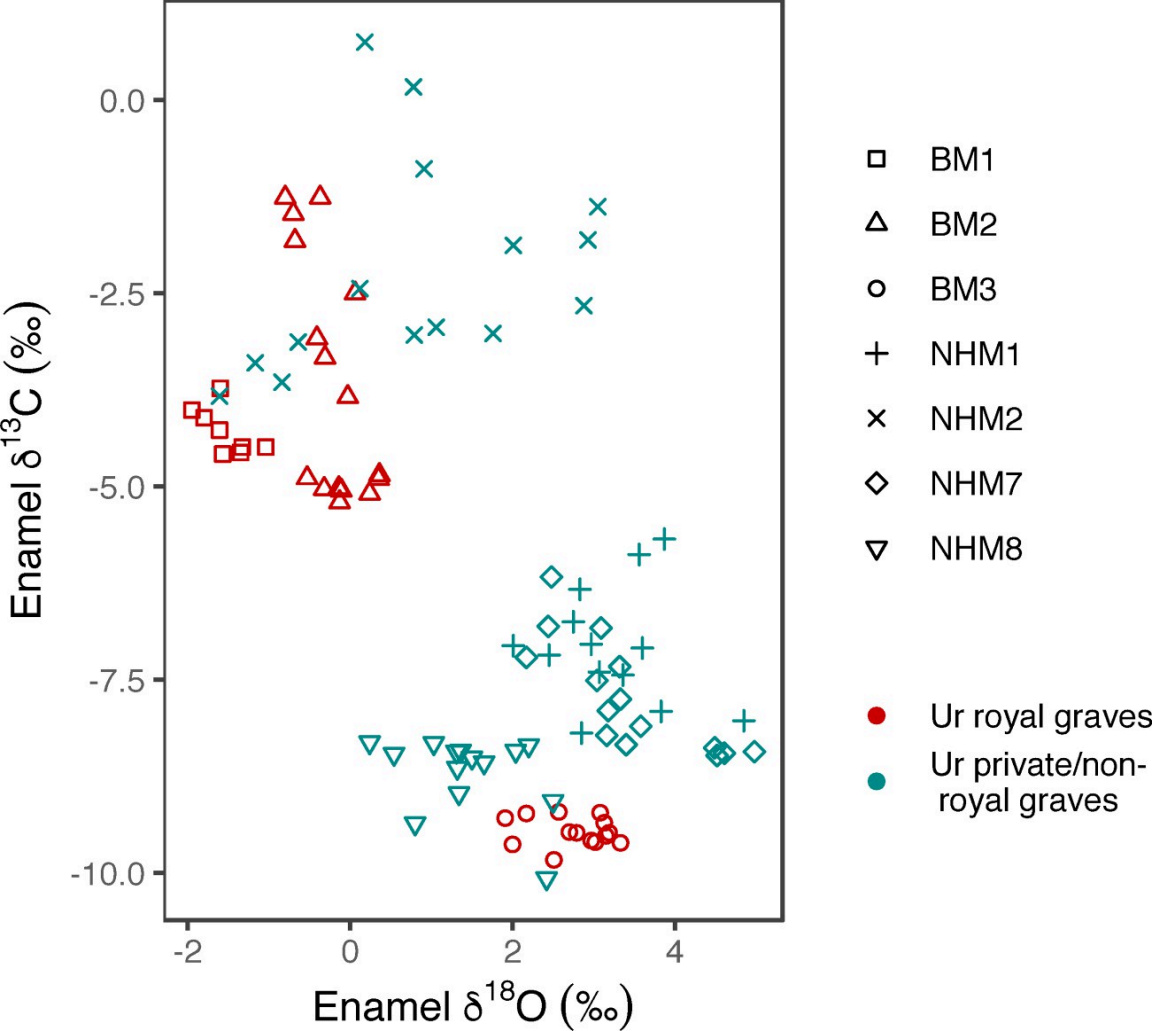
(bovid_OC.eps): Scatter plot of bovine enamel carbonate sub-sample carbon and oxygen isotope values, separated by individual (different shapes) and the royal/private/non-royal graves (different colours).

The patterning in the δ^13^C and δ^18^O values of the bovine teeth show two clusters (Fig 9) with animals from royal and non-royal contexts in each. There is a negative correlation between carbon and oxygen, indicating that those animals consuming more C_3_ plants have higher δ^18^O values, while those consuming more C_4_ plants have lower δ^18^O values. This pattern has been observed elsewhere and has been ascribed to two possible causes: plant physiological adaptations to arid conditions, leading to less evapotranspirative enrichment in C_4_ plant δ^18^O values or alternatively to grazing animals drinking more water [101–103].

## Discussion

### Mobility, water, food, and the Ur cattle

Our initial specific aim was to identify the degree of similarity and/or variability in the diet, water consumption and mobility patterns of bovines found in two distinct contexts at Early Dynastic III Ur: royal and private/non-royal graves. It might be expected that animals in royal graves had a more nutritious diet in comparison to private/non-royal animals, given that cattle offered to the gods are described as having been specially barley-fattened and physically perfect [104: 114, 105]. Animal offerings in royal graves might have been similarly selected and fed optimal diets from birth. However, the isotopic data demonstrate there was not a single separate ‘royal herd’ management strategy in evidence, rather that there were varied approaches to the management and movement of both royal and private/non-royal cattle.

It might be expected that the relatively high water requirements of cattle and their slow speed should mean minimal movement to and from grazing land, which would contrast to sheep and goats that predominantly would be grazed in areas further afield. If cattle did not move medium or long distances, they may have been fed via small-scale and local foddering, pasturing, and water arrangements, involving grazing in areas of land (e.g. fallow fields) adjacent to canals, drainage basins, or the river, within or close to the city. The enamel strontium isotopic data are consistent with both royal and private/non-royal cattle at Ur being raised on the southern Mesopotamian alluvial plain, with no indication of movement between isotopically different settings on a seasonal basis within the early years of their lives. However, from the strontium isotopic evidence, at least one bovine was moved to Ur from beyond the alluvial plain, indicating that the animals were not all locally raised. The enamel carbon isotopic data indicate a range of domestic cattle diets, with some animals displaying a predominantly C_3_ plant-based diet, and others a predominantly C_4_ plant-based diet. The enamel oxygen isotopic data indicate variable exploitation of plant and water sources, and this suggests a multi-faceted approach to moving cattle within and possibly beyond the city hinterland to find acceptable watering and grazing lands.

The main water sources in southern Mesopotamia during the third millennium BC include the Tigris and Euphrates Rivers, the marshes, and an extensive network of irrigation canals, drainage basins and inlet harbours, and while wells are known they probably provided for human water needs only [32,41,106–110]. All water sources in southern Mesopotamia can be subject to high evaporation rates that cause oxygen isotopic enrichment through the preferential removal of the light (H_2_^16^O) relative to the heavy (H_2_^18^O) isotopologues from standing bodies of water [71]. However, variable evaporation can be expected in different types of water source: limited in quickly-moving rivers, large canals, and the vast permanent marshes, versus higher evaporation in small field canals or temporary drainage basins. The high δ^18^O in bovine tooth enamel could result from animals drinking water from ^18^O-enriched sources, i.e. those subject to high evaporation, but might also be linked to consumption of different plant types.

Archaeobotanical and textual sources suggest that a range of plants were present in southern Mesopotamia, including various crops (primarily wheat and barley) and reeds [56]. Both C_3_ and C_4_ plants are present in the region, with C_4_ grasses and sedges being particularly well adapted to water stress (e.g., saline environs and arid conditions); most indigenous C_4_ plants in Iraq today are steppe and desert grasses and shrubs [111]. The plants that grow in the traditional wetlands of south Iraq include a range of different species of grasses, e.g., salt reeds (*Distichis*), which are adapted to arid environments and high temperatures [32: 14]. Significantly, marsh plants have variable photosynthetic pathways, with C_4_ plants including *Papyrus Cyperus*, and C_3_ plants including reedmace *(Typha angustfolia)*, rushes (*Juncus*), and common marsh reed (*Phragmites australis)*; such plants are common along the banks of canals of all sizes and in drainage basins, as well as in permanent and seasonal marshes [112,113]. Growing conditions affect plant isotopic values, both carbon and oxygen [114,115]. Water stress increases δ^13^C values. Increased evapotranspiration, which is not the same as water stress, increases plant δ^18^O values; this is commonly seen in species such as reeds growing in wetland environments.

Without comparative contemporary plant and water isotopic data, it is difficult to determine why the isotopic data from the Ur cattle is clustered into two groups: these groups could reflect consumption of different water sources, consumption of different plant types (C_3_ vs C_4_), differences in the growing conditions of plants (aridity, stress, evapotranspiration rates), or a combination of all these. However, we can highlight some likely possibilities, which have implications for our understanding of herd management strategies, and these include: foddering by reeds or barley grains and crop residues; pasturing on fallow fields; pasturing at the distal ends of irrigation systems, including the banks of minor canals, drainage canals or basins adjacent to steppe. The possibility that some animals were specially fattened with barley [104: 114] suggests that foddering was carried out. Reeds are currently used as cattle and water buffalo fodder in south Iraq, and reports from the early twentieth century suggest that this practice has some heritage [15, 32: 2, 113,116].

### Were there royal herds? implications for understanding herd management and mobility

An understanding of how animals were managed between the urban center of Ur and its surrounding hinterland is key information for a full reconstruction of Ur’s social organisation. The movement of herded animals within this small-scale landscape, as reflected by isotopic analyses, is a valuable contribution to this animal management picture. The data from Ur’s royal and private/non-royal graves has shown that there were some similarities and differences between these data from the two groups. As such, it does not appear that there was a definitive and single separate ‘royal herd’ management strategy in evidence. A number of scenarios for herd management might be conceived, placing control in the hands of royal and/or non-royal agents, and the use of isotope analysis makes it possible to differentiate different strategies. Despite the implications of texts, at least two different herding regimes were found in the ‘royal herd’ data, which likely made full use of the local alluvial plain and the potentials of the irrigation system. The patterns observed in these data suggest flexible animal management strategies at Ur, and that the animals that ended up in all types of graves in the Royal Cemetery were not kept separately during their lifetime. The presence of one animal that moved from farther afield (north or east) indicates that there was some longer-distance movement of animals, and it is notable that this animal was from a ‘royal’ grave.

While it is not currently possible to determine with complete certainty if all these samples were precisely contemporaneous, the data are suggestive of a variety of different simultaneous regimes, perhaps related to their owners’ differential access to types of land and water. If they are not contemporaneous, the isotopic differences could indicate subtle changes in the local environment and animal management practices at Ur over the short term.

The results from this study have already expanded our picture of Early Dynastic III human-animal interaction in southern Mesopotamia. The data from the isotope and trace element analyses suggests excellent potential for future studies that will explore the larger picture of the early Mesopotamian economy. Questions about micro-environments, climate change and animal management strategies can potentially be answered. This research represents the first results at exploring past relationships among humans, animals and the environment, including short distance mobility, and animal management. Observations have already begun to challenge long-standing theories on herd management and mobility, which builds a more comprehensive picture of third millennium BC society at Ur.

## Methods

### Sample selection

Faunal remains for royal and private/non-royal contexts were studied and identified to species or genus level, skeletal element, sex, age, pathologies, cultural modifications and taphonomic indicators; analyses are ongoing and results are pending. Well preserved cattle teeth were then selected for analysis of enamel δ^18^O, δ^13^C and ^87^Sr/^86^Sr analysis from royal (n = 4) and private/ non-royal (n = 4) contexts (see SI for Woolley’s grave designations). Fragments of shell (molluscs (bivalve) n = 11; (terrestrial snail) n = 1) were chosen for ^87^Sr/^86^Sr to gather baseline data. Domestic pig teeth (n = 2) were selected for δ^18^O, δ^13^C as comparative data.

### Sampling

For the teeth, the enamel surface was cleaned using mechanical abrasion. Powdered enamel samples were collected at discrete intervals along a transect from the occlusal surface to the enamel-root junction (ERJ). The entire depth of enamel was sampled and care was taken to avoid inclusion of any underlying dentine. Small fragments of mollusc shells were broken off of larger specimens used for ^87^Sr/^86^Sr analysis. There was no cleaning necessary prior to the dissolution of the specimens (see below).

### Sample preparation and analysis for δ^18^O and δ^13^C

Approximately 7mg of powdered enamel was prepared following Balasse, Ambrose [117]. Samples were treated with 2–3% aq. sodium hypochlorite solution (NaOCl) for 24 hours (0.1ml/mg sample), thoroughly rinsed with distilled water, treated with 0.1M acetic acid (CH_3_COOH) for 4 hours (0.1ml/mg sample), and thoroughly rinsed once more. δ^18^O and δ^13^C analysis was performed at the Godwin Laboratory, Department of Earth Sciences, University of Cambridge. Enamel powder samples were reacted with 100% orthophosphoric acid for 2 hours at 70°C in individual vessels in an automated Gasbench interfaced with a Thermo Finnigan MAT 253 isotope ratio mass spectrometer. These results were reported as delta values on the VPDB scale calibrated through the NBS19 standard [118] for which the precision is better than ±0.11‰ for ^18^O/^16^O and ±0.08‰ for ^13^C/^12^C.

## Sample preparation and ^87^Sr/^86^Sr analysis

Fragments of mollusc shell of less than 1 cm^2^ were dissolved in 0.5M HCl. Tooth enamel samples were treated with 0.1M acetic acid for 4 hours to remove any calcite that might be present in the samples [117]. Sr was purified from Ca and other elements using cation exchange chromatography in a Class 1000 laboratory equipped with Class 100 clean workstations following methods in [119]. The purified Sr was loaded on Ta single filaments and the isotopes of Sr measured on a Thermo-Elemental Triton instrument in the Saskatchewan Isotope Laboratory, Department of Geological Sciences, University of Saskatchewan, using a static multi-collection routine. The measured ^87^Sr/^86^Sr ratios were corrected for instrumental mass bias using the exponential law and 8.375209 for the isotopic ratio ^88^Sr/^86^Sr. The SRM 987 standard yielded 0.710268 ±0.000007 over the course of this work.

### Ur

The ancient city of Ur is located in southern Iraq, on the southern Mesopotamian alluvial floodplain c.16/17 km west of Nasiriyah [28,120: 167, 121]. Sir Henry Rawlinson [122: 47] was the first to identify Tell al-Muqayyar as ancient Ur, and consistently it has been identified as the biblical Ur [see: 123,124], and the birthplace of Abraham [121]. The British Museum and the University of Pennsylvania Museum conducted extensive excavations at Tell al-Muqayyar from 1922 until 1934, under the direction of Charles Leonard Woolley [28, 120: 167,^125^,^126^]. Woolley’s excavations showed that Ur became a major urban centre during the Early Dynastic period (c.2900-2400 BC).

The collection is currently divided between the British Museum (BM) and Natural History Museum (NHM) in London, with animals from the royal graves in the BM and assemblages from private/non-royal graves in the NHM. Grave determinations (royal or private/non-royal) were based on Woolley’s 1934 publication of the graves and his own designations [28]. Woolley’s faunal collections (both in the NHM and the BM) are the two largest assemblages from the early excavations at Ur. The faunal remains from the private graves is only a very small percentage (> 10%) of the total animal grave goods within the thousands of burials. The remains are also representative of what Woolley chose to ship home and hence potentially biased and not necessarily wholly demonstrative of consumption/ritual patterns of disposal.

### Abu Salabikh

Abu Salabikh is an Early Dynastic Period archaeological site to the north of Ur, also located on the Euphrates River during the third millennium [127]. The baseline geological data from an additional site in the region allowed for a greater breadth of information on local strontium levels.

### The cattle specimens

Samples of four cattle from royal burial contexts were obtained from the British Museum collections (Table 1), including three from PG 789 ‘King’s Grave’, and one from PG 800 ‘Pu-abi’s Grave’ (see: Woolley Text and Plates [28]) for grave locations and descriptions. Samples of four cattle from non-royal burial contexts were obtained from the Museum of Natural History collections (Table 1), including one each from Above PG 1054 (NHM1), unknown PG (NHM2), PG 15 (NHM3), and PG 144 (NHM4). (see [38], S6 Fig and, text below for grave locations and descriptions). All necessary permits were obtained for the described study, which complied with all relevant regulations. Both the NHM and the BM specimens were sampled under the approval of their scientific committees for each institution; The Natural History Museum, London, SW7 5BD “Destructive and Invasive Sampling Request Form”, and for the British Museum, Great Russel Square, London, WC1B 3DG “Application to Conduct Scientific Analysis/Examination of British Museum Collection Material–Form EE1”. Applications to perform scientific analyses on the teeth were submitted with full documentation (pictures, measurements, zooarchaeological identification, and conservation methods, etc.) of the process required to complete the data extraction from the teeth.

Archaeological contexts for each specimen:

PG789 is determined to be royal based on a number of artifacts/monumental domed mud-brick burial chamber, and this is the designation that Woolley provided (S1–S4 Figs). While some scholars have debated the owner of the tomb, its designation as Royal has not been challenged. In fact, recent scholarship maintains that this tomb appears to be that of King Meskalamdug [29: 96].

**SI_Fig_1** PG789 The Great Death Pit 789. Republished from [Ur-online.org cf. Woolley 1934 Vol. II Plate 30] under a CC BY license, with permission courtesy of the Penn Museum original copyright 1934.

**SI_Fig_2** PG 789 layout of tomb. Republished from [Ur-online.org cf. Woolley 1934 Vol. II Plate 29] under a CC BY license, with permission courtesy of the Penn Museum original copyright 1934.

**SI_Fig_3** PG789 image of an entire *insitu* cattle specimen. Republished from [Ur-online.org cf. Woolley 1934 Vol. II Plate 35a] under a CC BY license, with permission courtesy of the Penn Museum original copyright 1934.

**SI_Fig_4** PG789 close up images of two *insitu* cattle cranium. Republished from [Ur-online.org cf. Woolley 1934 Vol. II Plate 35b] under a CC BY license, with permission courtesy of the Penn Museum original copyright 1934.

PG800 Referred to as one of the Great Death Pits within the Royal Cemetery, this tomb was Queen Pu-abi’s and filled with elite grave goods including a cylinder seal with her name on it, numerous attendants (both men and women), and cattle still harnessed to their cart (S5 Fig). Based on the artifacts recovered in association with the chamber tomb architecture, Woolley designated this grave as one of the Royal Tombs within the cemetery [28,29: 108].

The tooth chosen (BM3) 1928.10.10,164 is from Pu-abi’s Grave for this study appears backwards in the picture (see Fig 6 in text). Decades prior to this study it was erroneously placed in backwards by BM conservators when the specimen was mounted for display. The ‘asses’ (assigned by Woolley) that were attached to the collar have since been correctly identified as cattle [Ur-online.org cf. Woolley’s 1934 Vol. II Plate 39 for further images of the specimen *insitu* with harness].

**SI_Fig_5** PG800 layout of tomb. Republished from [Ur-online.org cf. Woolley 1934 Vol. II Plate 36] under a CC BY license, with permission courtesy of the Penn Museum original copyright 1934, image 53678.

PG‘Above 1054’ (NHM1)—Woolley labeled this specimen as coming from the ‘burnt layer above 1054’. We have to presume this specimen was not part of the inner chamber of 1054 otherwise it would have a specific 1054 Royal Tomb label. This designation (see Table 1: burnt layer above 1054) illustrates this context.

PGUnknown (NHM2)—The ‘unknown’ specimen is from a private grave based on the tag that Woolley wrote for this specimen. If there had been a royal designation for this deposit, presumably it would have been noted on the tag and collected at the same time as the animal bones from the royal tombs. Based on the rest of the faunal assemblage associated with this specimen, it appears that this specimen was part of a partial, but articulated, mandible and maxilla. It probably represented a grave offering similar to those found within the other non-royal graves examined where only a portion of an animal body was buried versus a full skeleton as those found within the royal grave goods.

PG15 (NHM3)—Woolley’s tag states clearly that PG 15 is a private grave from the “foundation path” and is designated as a grave from the general Pre-Dynastic period (B). It was not designated as a royal tomb from the earliest part of the period (A). See [28, Appendix A: 412]. Grave type L (larnex or clay coffin). This is not the poorest type of grave in the cemetery, but it is at the poorer end of the wealth scale (e.g., grave goods) and cannot be considered a royal tomb (or perhaps even elite). PG15 has a cylinder seal (U7657), a pin and a bead [28, Appendix A: 412].

PG144 (NHM4)—This non-royal PG grave is assigned to the B category (non-royal)–it belongs to the Pre-Dynastic (Early Dynastic) cemetery. There is a lack of a domed chamber and very little grave goods (pin, 3 vessels, animal teeth) in relation to the finds from the royal tombs (see Woolley [28, Appendix A: 416–7]. In addition, it has a Grave type T designation (trench burial–simple internment wrapped in matting) [28, Appendix A: 411].

**SI_Fig_6** Before and after drilling images of specimens from the Natural History Museum (NHM#s 1–4), chosen for this study (Reade 2014).

The Shell Specimens

All of the snail shells and mollusc specimens from Ur and Abu Salabikh are from secure archaeological deposits dated to the Early Dynastic Period.

## Supporting information

S1 Fig PG789 The Great Death Pit 789 Republished from [Ur-online.org cf. Woolley 1934 Vol. II Plate 30] under a CC BY license, with permission courtesy of the Penn Museum original copyright 1934. (TIF)

S2 Fig PG 789 layout of tomb Republished from [Ur-online.org cf. Woolley 1934 Vol. II Plate 30] under a CC BY license, with permission courtesy of the Penn Museum original copyright 1934. (TIF)

S3 Fig PG789 image of an entire cattle specimen *insitu*. Republished from [Ur-online.org cf. Woolley 1934 Vol. II Plate 35a] under a CC BY license, with permission courtesy of the Penn Museum original copyright 1934.
(TIF)

S4 Fig PG789 close up images of two cattle cranium *insitu*. Republished from [Ur-online.org cf. Woolley 1934 Vol. II Plate 35b] under a CC BY license, with permission courtesy of the Penn Museum original copyright 1934.
(TIF)

S5 Fig PG800 layout of tomb. Republished from [Ur-online.org cf. Woolley 1934 Vol. II Plate 36] under a CC BY license, with permission courtesy of the Penn Museum original copyright 1934.
(TIF)

S6 Fig Before and after drilling images of specimens from the Natural History Museum (NHM#s 1–4), chosen for this study (Reade 2015). (TIF)

S1 Table (UrAbS_all_dataR.csv): Data are retrieved from eight bovine teeth ^87^Sr/^86^Sr data, and δ^13^C and δ^18^O from seven bovines. Twelve molluscs provided ^87^Sr/^86^Sr data included in this table.
(CSV)

S1 File RCode: UrAbS_codejan2021.R. (R)
