# Supplementary figures and images for: Were there royal herds? Understanding herd management and mobility using isotopic characterizations of cattle tooth enamel from Early Dynastic Ur
Source: PLoS One. 2022 Jun 15;17(6):e0265170. doi: 10.1371/journal.pone.0265170 (PMC9200365; doi:10.1371/journal.pone.0265170)

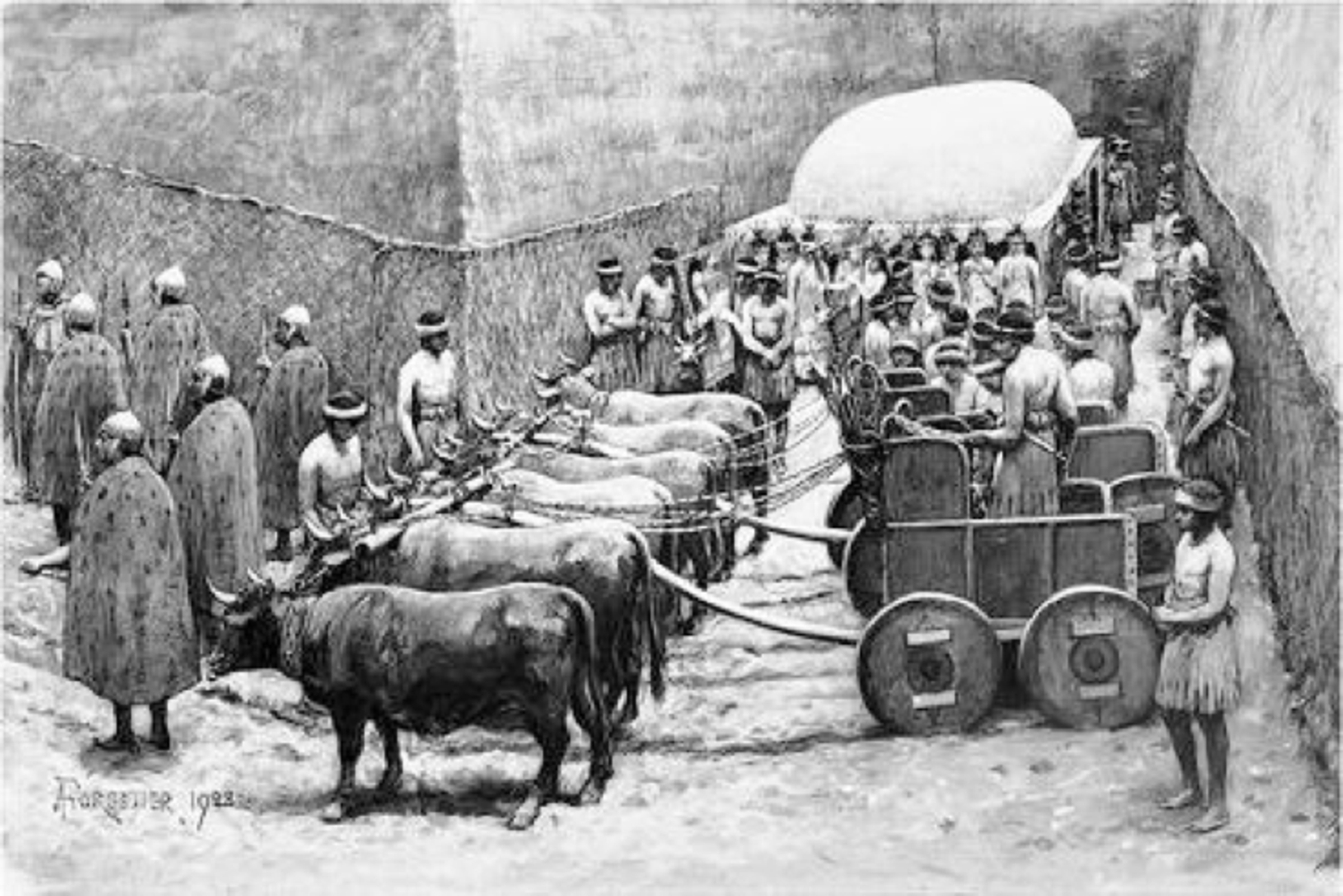

Supplement: S1 Fig — (TIF) [file pone.0265170.s001.tif]

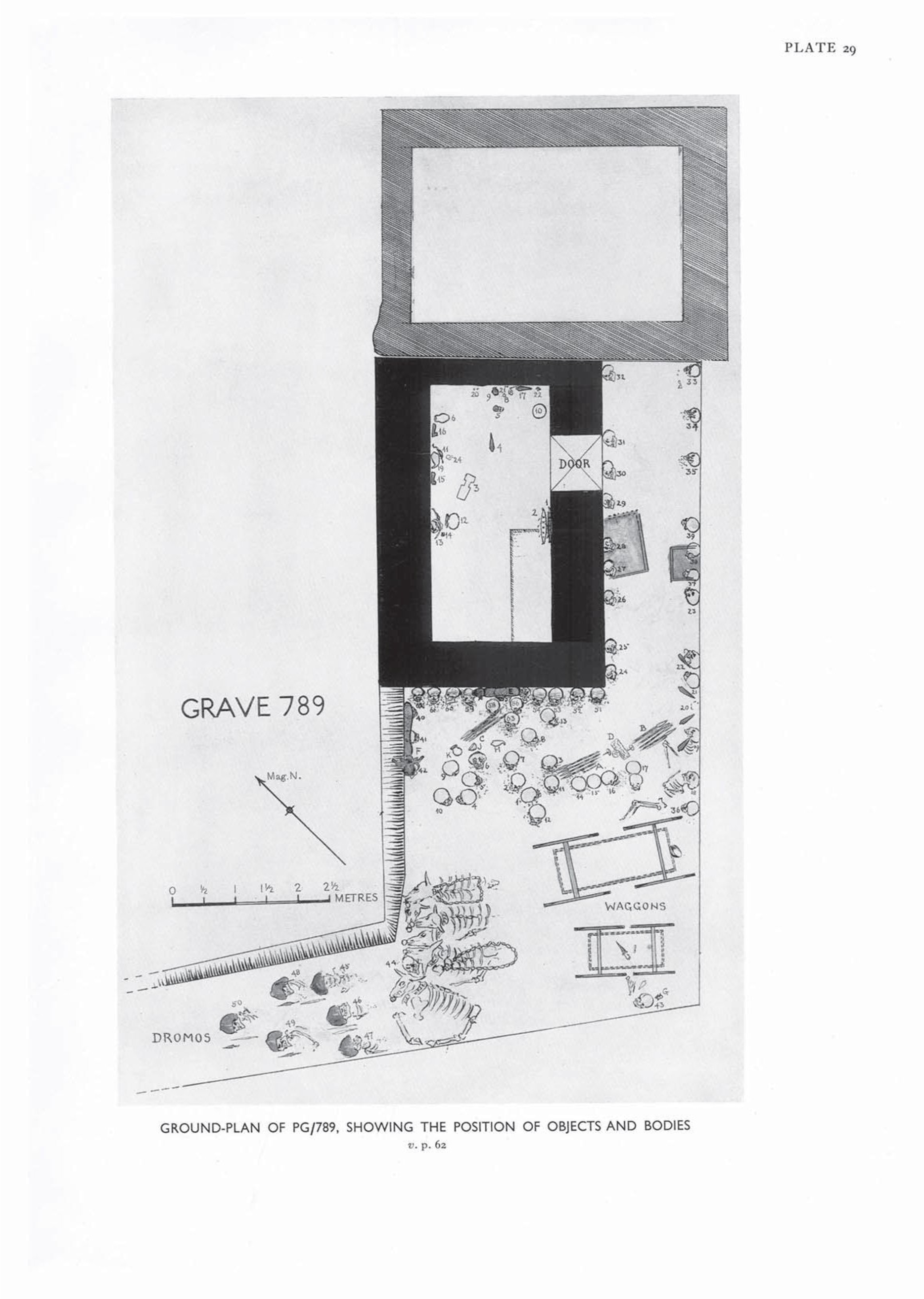

Supplement: S2 Fig — (TIF) [file pone.0265170.s002.tif]

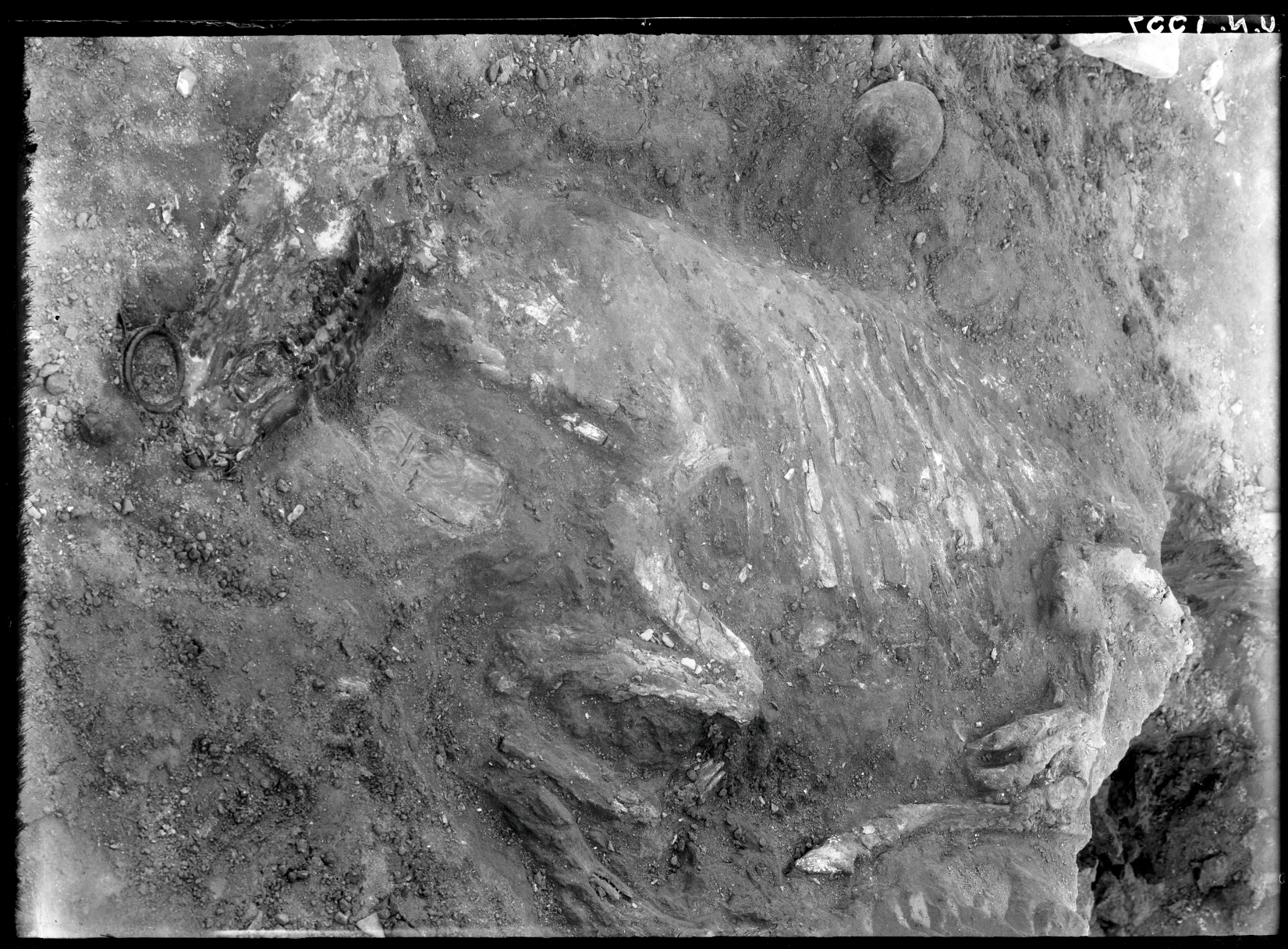

Supplement: S3 Fig — Republished from [Ur-online.org cf. Woolley 1934 Vol. II Plate 35a] under a CC BY license, with permission courtesy of the Penn Museum original copyright 1934. (TIF) [file pone.0265170.s003.tif]

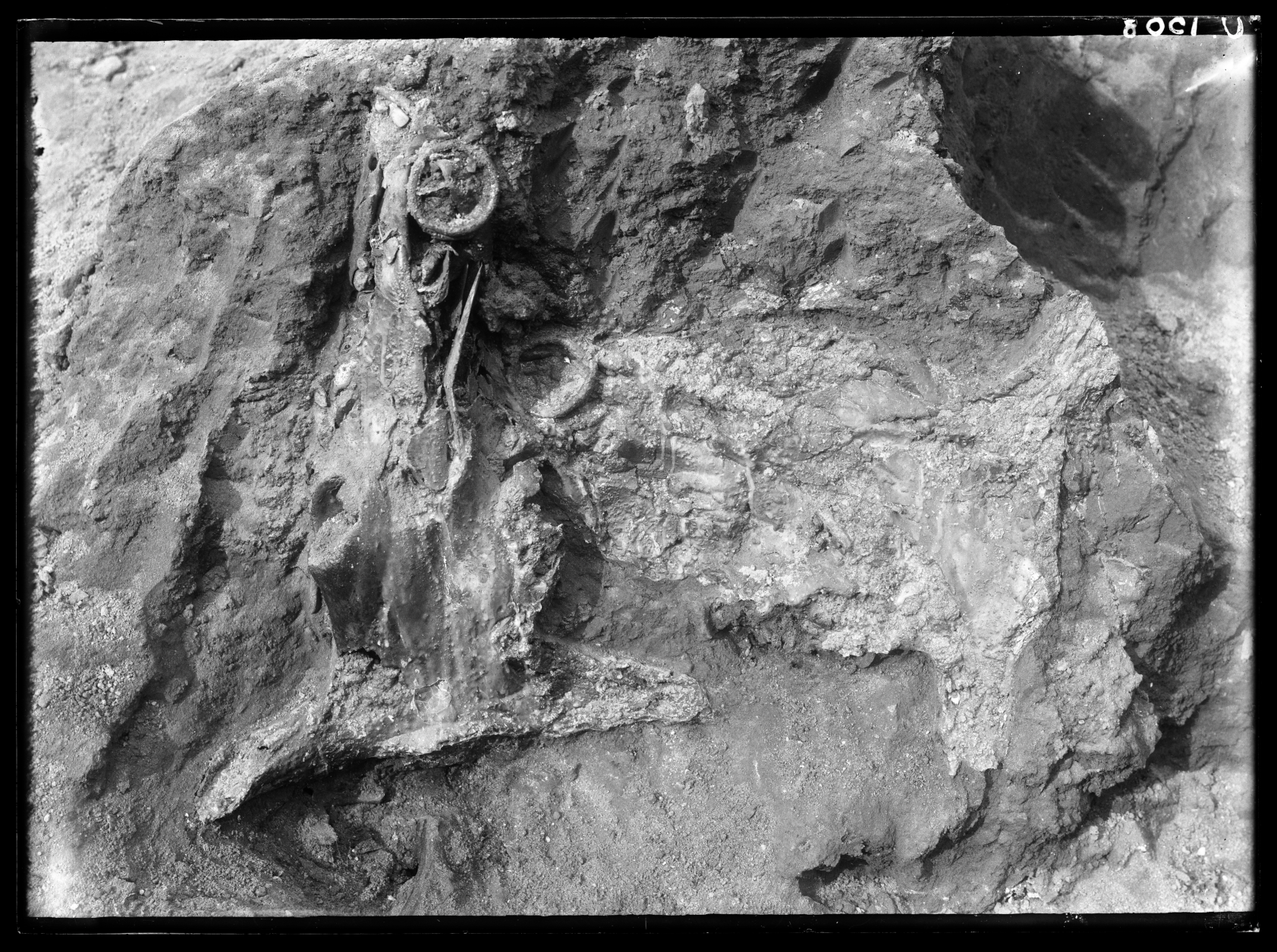

Supplement: S4 Fig — Republished from [Ur-online.org cf. Woolley 1934 Vol. II Plate 35b] under a CC BY license, with permission courtesy of the Penn Museum original copyright 1934. (TIF) [file pone.0265170.s004.tif]

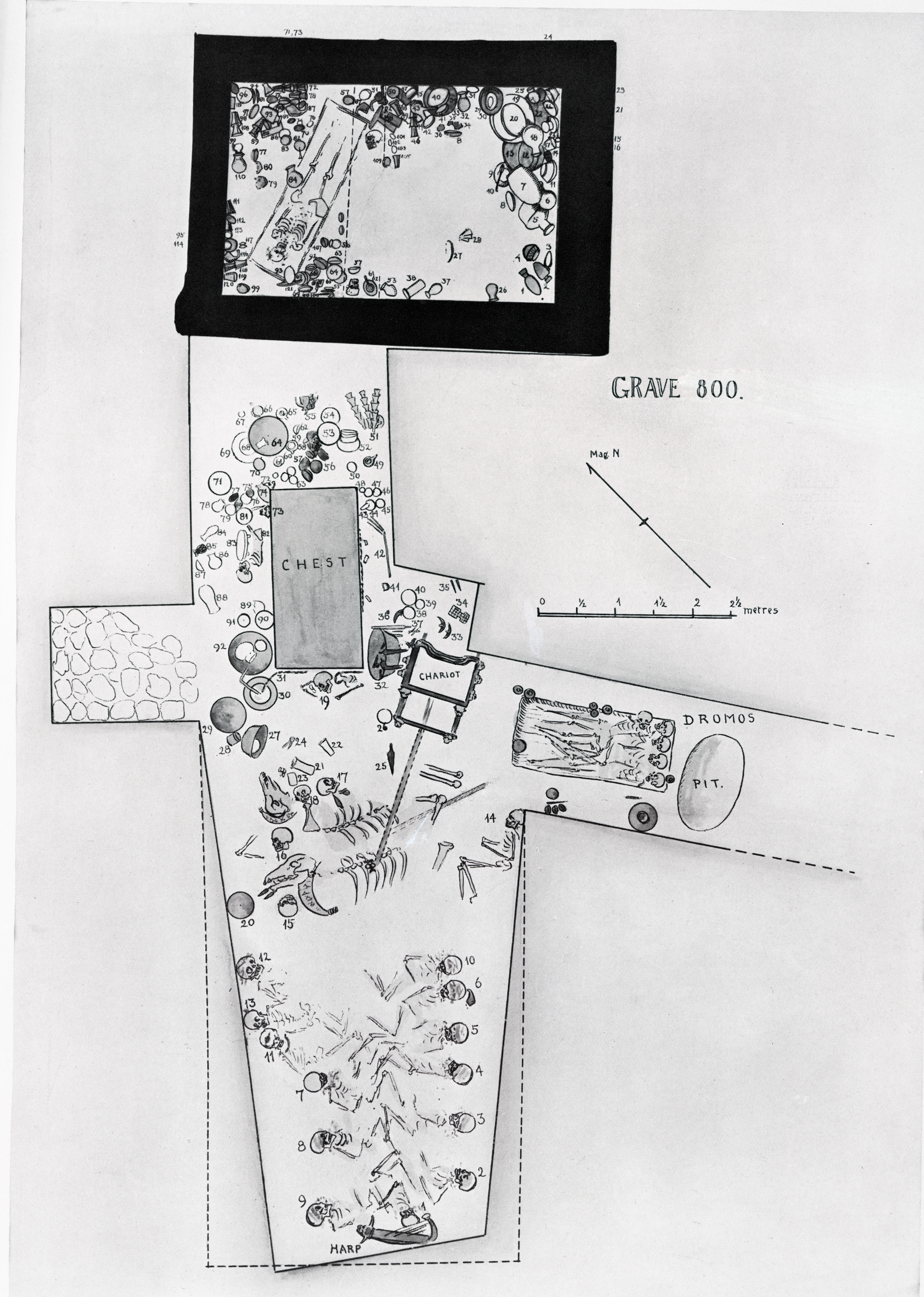

Supplement: S5 Fig — Republished from [Ur-online.org cf. Woolley 1934 Vol. II Plate 36] under a CC BY license, with permission courtesy of the Penn Museum original copyright 1934. (TIF) [file pone.0265170.s005.tif]

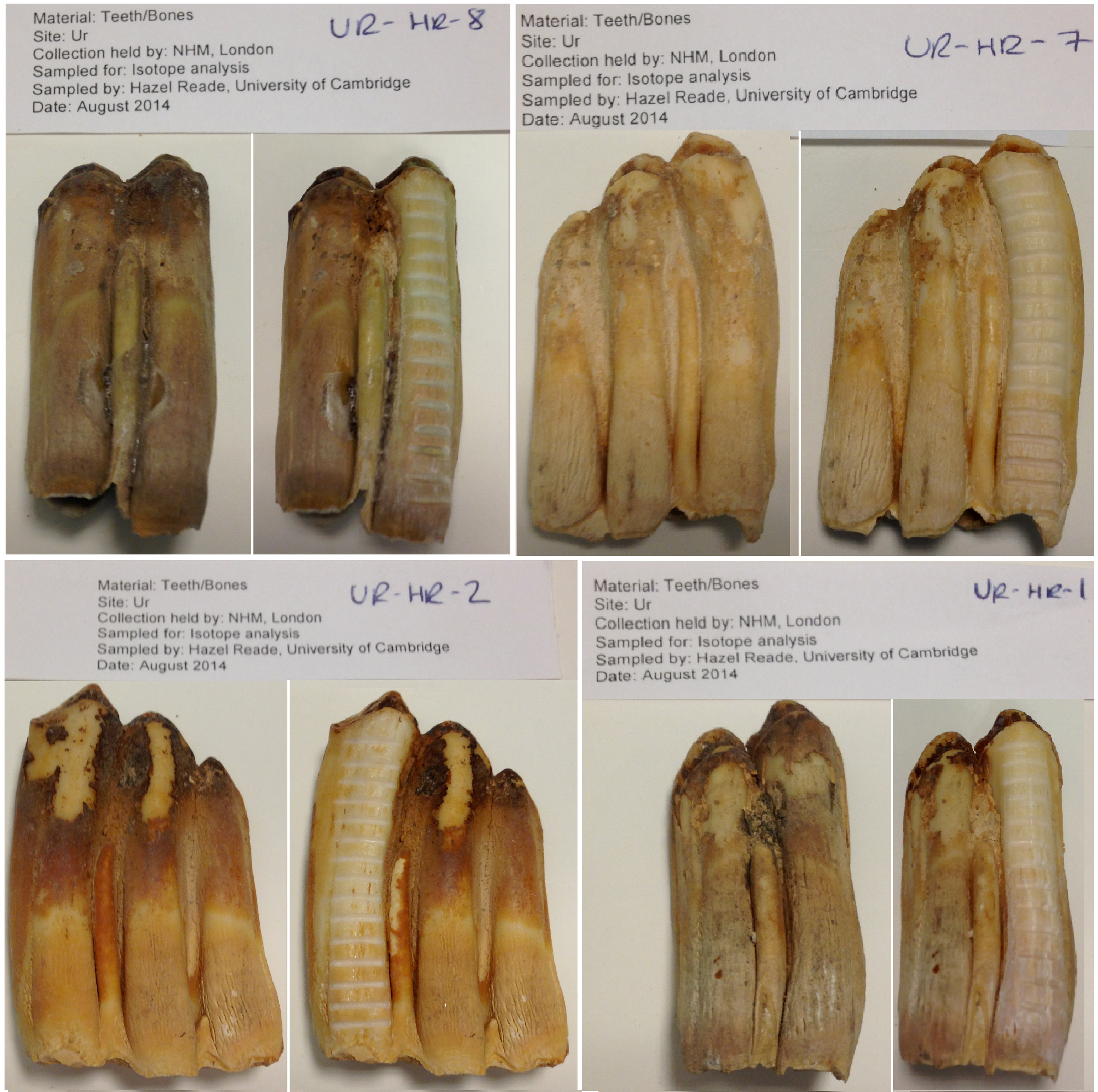

Supplement: S6 Fig — (TIF) [file pone.0265170.s006.tif]
